# Discovery of Fe_7_O_9_: a new iron oxide with a complex monoclinic structure

**DOI:** 10.1038/srep32852

**Published:** 2016-09-08

**Authors:** Ryosuke Sinmyo, Elena Bykova, Sergey V. Ovsyannikov, Catherine McCammon, Ilya Kupenko, Leyla Ismailova, Leonid Dubrovinsky

**Affiliations:** 1 Bayerisches Geoinstitut, Universität Bayreuth, D-95440 Bayreuth, Germany; 2 Laboratory of Crystallography, Universität Bayreuth, D-95447 Bayreuth, Germany; 3 European Synchrotron Radiation Facility, BP220, F-38043 Grenoble, France

## Abstract

Iron oxides are fundamentally important compounds for basic and applied sciences as well as in numerous industrial applications. In this work we report the synthesis and investigation of a new binary iron oxide with the hitherto unknown stoichiometry of Fe_7_O_9_. This new oxide was synthesized at high-pressure high-temperature (HP-HT) conditions, and its black single crystals were successfully recovered at ambient conditions. By means of single crystal X-ray diffraction we determined that Fe_7_O_9_ adopts a monoclinic *C2/m* lattice with the most distorted crystal structure among the binary iron oxides known to date. The synthesis of Fe_7_O_9_ opens a new portal to exotic iron-rich (*M*,Fe)_7_O_9_ oxides with unusual stoichiometry and distorted crystal structures. Moreover, the crystal structure and phase relations of such new iron oxide groups may provide new insight into the cycling of volatiles in the Earth’s interior.

Iron oxides have great importance for all natural sciences as well as numerous industrial applications. Considering the high abundance of oxygen and iron in the Earth’s crust and the mantle, binary iron oxides and their derivatives are important endmembers of phases that make a significant contribution to properties of the Earth. Many studies have been devoted to investigations of various properties of iron oxides at conditions relevant to the Earth’s interior, i.e., at high pressure or at high pressure combined with high temperature (HP-HT). These studies reported a number of remarkable findings for the three basic iron oxides, Fe_1-x_O wüstite^1^,^2^, *α*-Fe_2_O_3_ hematite^3^,^4^,^5^,^6^,^7^,^8^, and Fe_3_O_4_ magnetite^9^,^10^,^11^. Meanwhile, several experimental and theoretical studies indicated that the chemistry of iron oxides at extreme conditions of high pressure and high temperature may extend to intriguing behavior beyond these three well-known oxides^12^,^13^,^14^,^15^. For instance, recent experimental HP-HT investigations in the pressure range of 10–20 GPa reported two new orthorhombic *Cmcm* polymorphs with Fe_4_O_5_^13^ and Fe_5_O_6_^14^ stoichiometry, i.e., between Fe_3_O_4_ and Fe_1-x_O, and hence, with mixed Fe^2+^ and Fe^3+^ oxidation states. More interestingly, using modern theoretical methods an opposite tendency in the stoichiometry changes in Fe-O systems was found, suggesting an extended stability of ferric hematite and a gradual shift to Fe^4+^–bearing oxides at megabar pressures^15^. In particular, these studies predicted the existence of a new exotic FeO_2_ oxide with highly charged Fe^4+^ ions that should have an extended range of stability against decomposition under megabar pressures^15^. Recently, a number of experimental studies have shown the important role of the iron oxides under HP-HT conditions in the Earth’s deep interior^16^,^17^,^18^,^19^. Decomposition reactions of iron oxides and oxyhydroxides may induce the release of oxygen or hydrogen in the Earth’s lower mantle^17^,^18^.

In addition to their obvious and primary importance for geosciences, iron oxides play crucial roles in many technological processes and applications, and remain among a handful of key materials with significant impact on the fundamental behavior of materials, including charge carrier transfer and interactions, spin dynamics of electrons, and other central topics. In other words, iron oxides are important prototype materials. For instance, the oldest-known natural magnet, magnetite, demonstrates a fascinating ‘metal-insulator’-type transition near 120–125 K^20^, named later as the ‘Verwey transition’ after its discovery in 1939. This Verwey transition was believed to be related to charge ordering on octahedral sites in the spinel structure, and has been hotly debated for decades. Only recently it was revealed that this transition is linked to formation of hitherto unknown ‘quasiparticles’ consisting of three bonded Fe ions called ‘trimerons’^21^. In hematite another landmark transition was discovered near 255 K with a related abrupt and drastic reorientation of spins of Fe^3+^ ions, named afterwards as a ‘spin-flop’ or Morin transition^22^. Also iron-deficient wüstite, Fe_1-x_O, displays puzzling complexities with regard to stoichiometry, defect structure, and elastic and physical properties^23^,^24^,^25^,^26^,^27^,^28^, and serves as a prototype for systems with non-stoichiometry. Recently it was experimentally demonstrated that, similar to magnetite, the newly-discovered Fe_4_O_5_^13^ also undergoes an unprecedented ‘metal-insulator’–type transition upon cooling below 150 K with competing dimeric and trimeric ordering in the Fe chains, leading to strong structural modulations^29^. Therefore, synthesis of new iron oxides with both mixed Fe^2+^ and Fe^3+^ valences and bearing highly charged Fe^4+^ ions is of significant interest for many scientific fields.

In the present work we investigated the phase stability of iron oxides at HP-HT conditions (see Methods) and discovered in samples recovered at ambient conditions the presence of crystals of a new iron oxide with unusual Fe_7_O_9_ stoichiometry. Fe_7_O_9_ with its ratio of Fe/O ~ 0.777 lies between Fe_3_O_4_ and newly-discovered Fe_4_O_5_^13^, but in contrast to both, it is non-magnetic at ambient conditions and adopts a monoclinic crystal structure with four sites for Fe cations. These observations combined with the difference in Fe^3+^/Fe^2+^ ratios (^4^/_3_ in Fe_7_O_9_ *versus* 2 in Fe_3_O_4_ and 1 in Fe_4_O_5_) suggest that the physical properties of Fe_7_O_9_ may be remarkably different. In addition, we also synthesized Fe_7_O_9_ containing a significant amount of Mg and discuss possible geological implications for this new polymorph.

## Results and Discussion

### Single crystal XRD measurements

The single crystals of pure Fe_7_O_9_ and Mg-doped (Mg,Fe^2+^)_3_Fe^3+^_4_O_9_ had sizes less than 50 μm in their linear dimensions that restricted detailed investigations of their properties. The chemical composition of the samples was determined using conventional electron microprobe methods and from single crystal X-ray diffraction data. We also collected Mössbauer spectra from these samples to determine the oxidation states of the Fe ions.

**Figure 1:**
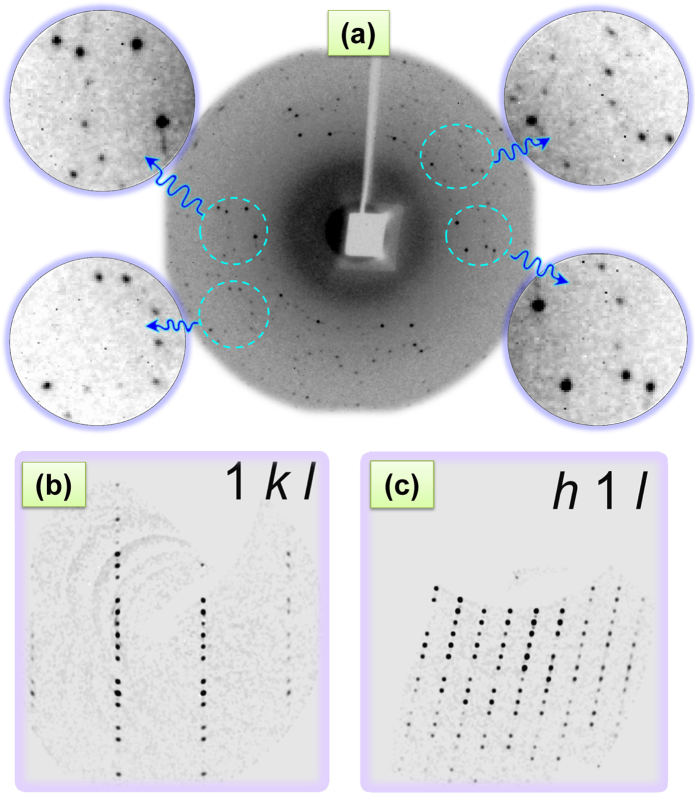
X-ray diffraction images. (**a**) Example of X-ray diffraction image of microscopic single crystal of (Mg,Fe)_3_Fe_4_O_9_ collected at ambient conditions under rotation of a sample over 360 degrees in a beam. This image contains several hundreds of small well-resolved reflections. Insets show selected magnified areas of this image that better show indistinct reflections. (**b**,**c**) Projections of these X-ray diffraction data in reciprocal space, for two selected planes, *1kl* (**b**) and *h1l* (**c**).

**Figure 2:**
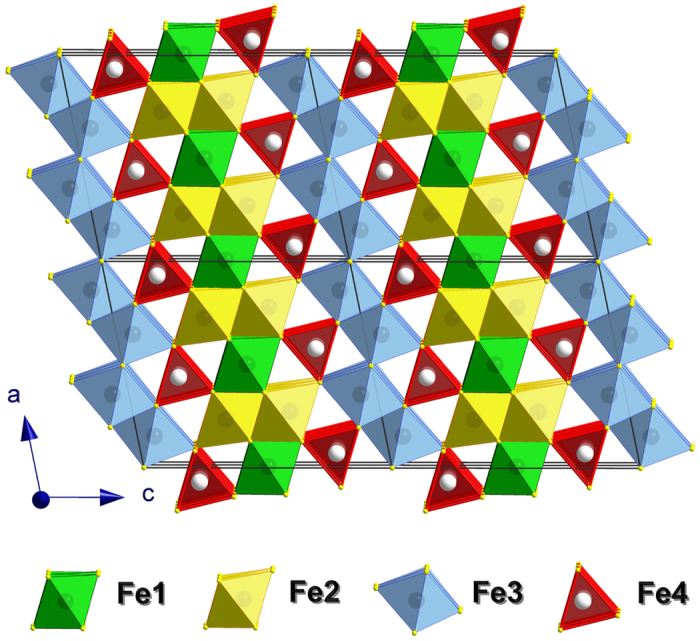
The crystal structure of Fe_7_O_9_. This structure corresponds to ambient conditions and is shown in a projection along the *b* axis.

By means of single crystal X-ray diffraction on the crystals (Fig. 1 and Supplementary Figure 1) we solved and refined the crystal structures of Fe_7_O_9_ and (Mg,Fe^2+^)_3_Fe^3+^_4_O_9_ at ambient conditions and confirmed their stoichiometry. Crystals of Fe_7_O_9_ and (Mg, Fe^2+^)_3_Fe^3+^_4_O_9_ had no pronounced asymmetry in their shape, but were rather small (0.03 × 0.02 × 0.01 and 0.03 × 0.02 × 0.02 mm^3^), so it was not possible to perform an analytical absorption correction based on crystal shape. Crystals of Fe_7_O_9_ appeared to be twinned, and due to the high degree of peak overlap (>50%), we had to integrate both twin domains simultaneously to perform a refinement of the crystal structure against HKLF5 data (BASF value was about 49.8%). The twinning could also influence the quality of determination of the anisotropic parameters. For consistency, we also refined the structure in an isotropic approximation and demonstrated a negligible influence on the atomic positions (CIF-files in the Supplementary Materials). Technical details of the structure determinations are summarized in Tables S1 and S2 in the Supplementary Materials. We established that both compounds adopt the same monoclinic structure of the *C*2/*m* space group. The unit cell parameters in Fe_7_O_9_ are as follows: *a* = 9.696(2) Å, *b* = 2.8947(6) Å, *c* = 11.428(3) Å, *β* = 101.69(2)°, *V* = 314.10(12) Å^3^, and *Z* = 2 (Fig. 2, and Tables S1 and S2 and CIF-files in the Supplementary Materials). The crystal structure of Fe_7_O_9_ has four different crystallographic sites for cations, three (Fe1, Fe2, Fe3) are octahedrally–coordinated and connected in a 3D network, while the fourth, Fe4, has a trigonal-prismatic arrangement (Fig. 2 and Table S2 in the Supplementary Materials). By analyzing the Fe-O bond distances in this polymorph using a bond valence sums (BVS) method^30^, we established the average oxidation state for Fe ions occupying the Fe1, Fe2, Fe3, and Fe4 sites to be +2.74, +2.72, +2.82, and +2.10, respectively. Thus, we conclude that the Fe4 sites are occupied almost exclusively by Fe^2+^ ions (Fig. 2). The other octahedral sites participate in electronic exchange between Fe^2+^ and Fe^3+^ ions through the polaron hopping mechanism, similar to the octahedral network of magnetite^20^ and other Fe-bearing oxides^31^. Thus, the BVS method shows the average charge for each of the Fe1-Fe3 sites. We note that various considerations lead to octahedral sites in the recently-discovered Fe_4_O_5_ phase having different charges^29^.

Mg-doped Fe_7_O_9_ crystals adopt the same crystal structure as Fe_7_O_9_ with similar unit cell parameters: *a* = 9.6901(12) Å, *b* = 2.8943(5) Å, *c* = 11.4397(15) Å, *β* = 102.045(14)°, *V* = 313.77(8) Å^3^, and *Z* = 2 (Fig. 2 and Table S1 in the Supplementary Materials). Electron microprobe analysis established their chemical composition to be Mg_1.06_Fe_5.94_O_9_, i.e., nearly 15% of Fe ions are substituted by Mg. We used this chemical composition in the crystal structure refinement and found that Mg^2+^ ions occupy all four Fe sites, but with a noticeable preference for (*i*) the spacious Fe4 sites that are occupied by the larger Fe^2+^ ions in Fe_7_O_9_, and (*ii*) the Fe1 sites, located between the Fe4 sites (Fig. 2, Table S2 in the Supplementary Materials). In the case of Mg doping of Fe_4_O_5_, Mg ions were also found to occupy all Fe sites in the structure^32^. Repeating the same BVS analysis^30^ as above for Fe_7_O_9_ taking into account the Mg atom distribution determined by single crystal X-ray diffraction (Table S2 in the Supplementary Materials), we confirmed the ferrous nature of Fe4 ions and detected a minor increase in the BVS of all other octahedrally coordinated Fe ions as +2.81, +2.78, and +2.87 for the Fe1, Fe2, and Fe3 sites, respectively (Fig. 2). Thus, the results of BVS analysis suggest the persistence of charge transfer in the 3D octahedral network with the distributed Mg ions. However, these Mg impurities should dramatically lower the mobility of hopping polarons, and hence, the bulk electrical conductivity of Mg-doped Fe_7_O_9_ is expected to be much lower than that of Fe_7_O_9_.

### Mössbauer spectroscopy

**Figure 3:**
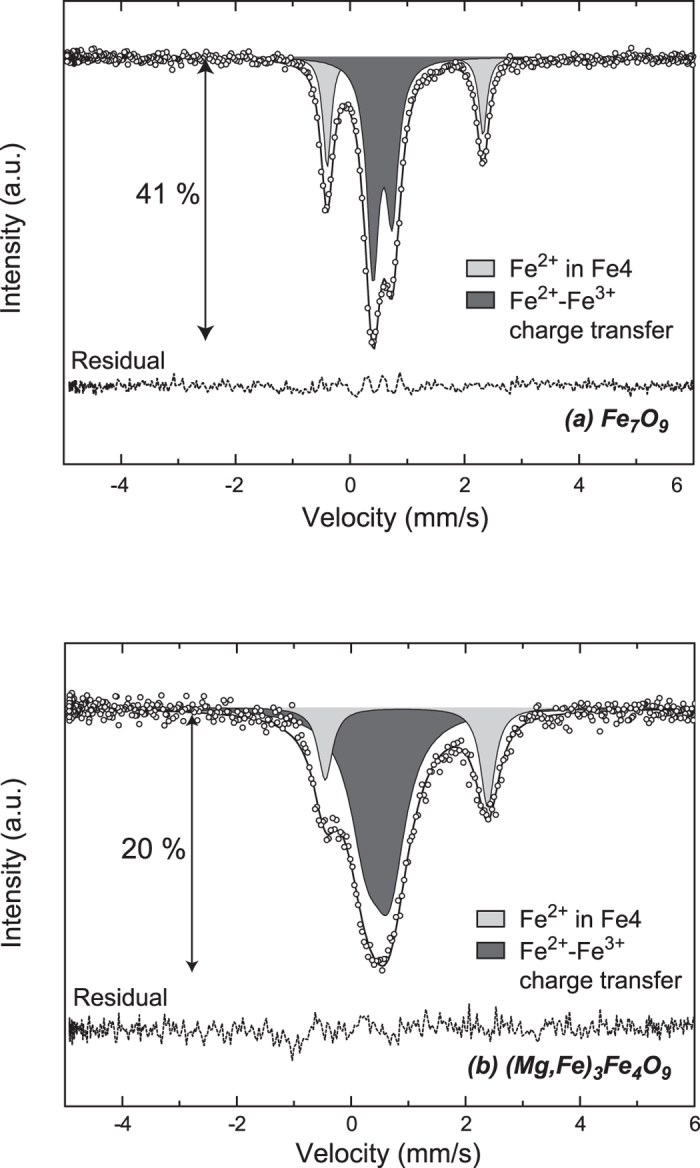
Mössbauer spectra of Fe_7_O_9_ samples. (**a**) Single crystal of Fe_7_O_9_. (**b**) Single crystal of (Mg,Fe)_3_Fe_4_O_9_. Both spectra were collected at ambient conditions. Black open circles, experimental spectrum; lines, fitted spectra; broken line, residual.

Both Fe_7_O_9_ and (Mg,Fe)_7_O_9_ samples were analyzed by Mössbauer spectroscopy using a synchrotron Mössbauer source that gave excellent signal to noise ratios (Fig. 3 and Table S3 in the Supplementary Materials). We did not observe any magnetic component in these spectra, and hence conclude that these materials are non-magnetic at ambient conditions. The spectrum of Fe_7_O_9_ (Fig. 3a) could be fitted by a superposition of two basic components, including (*i*) Fe^2+^ ions at the prismatically-coordinated Fe4 sites in the crystal structure (Fig. 2) and (*ii*) a merged component related to octahedrally-coordinated Fe1, Fe2, and Fe3 ions with an average oxidation state of Fe^2.8+^ (Fig. 3a and Table S3 in the Supplementary Materials). We note that this Fe^2.8+^ component is an average because of the above-mentioned rapid charge exchange between Fe^2+^ and Fe^3+^ ions at the octahedral sites, similarly to Fe_3_O_4_^33^. This finding is in excellent agreement with the above BVS oxidation states of the Fe ions obtained from the single crystal XRD data. It should be noted here that the three slightly structurally-inequivalent Fe1, Fe2 and Fe3 sites (Fig. 2) give similar contributions to the Mössbauer spectrum because of the very similar environment (edge-sharing FeO_6_ polyhedron) of the Fe ions, so it is not possible to separate their individual components in the spectrum (Fig. 3a). Spectra obtained from (Mg,Fe)_7_O_9_ were quite similar to Fe_7_O_9_ (Fig. 3b), while the merged component in the Mg-bearing sample spectrum shows a slightly higher average oxidation state of Fe^2.9+^ due to incorporation of Mg.

## Discussion

**Figure 4:**
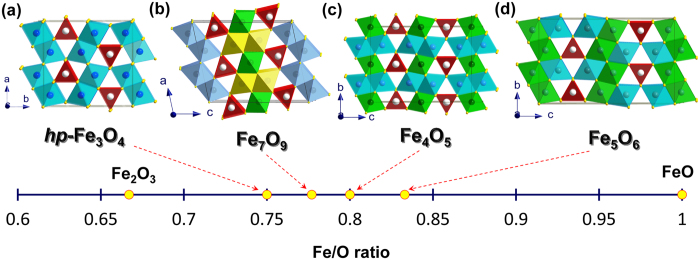
Comparison of unit cells of crystal structures of iron oxides. (**a**) High-pressure orthorhombic polymorph of Fe_3_O_4_^34^, (**b**) Monoclinic Fe_7_O_9_ polymorph discovered in the present work. (**c**) Orthorhombic *Cmcm* polymorph of Fe_4_O_5_ discovered in ref. 13. (**d**) Orthorhombic *Cmcm* polymorph of Fe_5_O_6_ discovered in ref. 14. Different colors of the octahedra denote different crystallographic sites for Fe ions.

In our work we synthesized Fe_7_O_9_ and (Mg,Fe^2+^)_3_Fe^3+^_4_O_9_ crystals at high pressures around 24–26 GPa. In previous studies the orthorhombic polymorphs of Fe_4_O_5_^13^ and Fe_5_O_6_^14^ were prepared at substantially lower pressures, between 10 and 20 GPa. It is interesting to note that the first pressure-driven structural phase transitions in the known iron oxides were detected at similar pressures around 20–25 GPa. For instance, at room temperature cubic Fe_1-x_O wüstite with the rocksalt structure transforms to a rhombohedral lattice above 20 GPa^1^, while at high temperatures the rocksalt structure of Fe_1-x_O is stable to at least 60 GPa^1^,^2^. High temperature-assisted phase transitions in Fe_3_O_4_ from cubic spinel to an orthorhombic phase^34^,^35^, and from corundum-type *α*-Fe_2_O_3_ to an orthorhombic Rh_2_O_3_(II)-type or to other phases^4^,^5^,^6^,^7^,^8^ were also observed in some studies already above 20–25 GPa, although these phase transitions are still hotly debated. These observations suggest that all conventional iron oxides (*α*-Fe_2_O_3_, Fe_3_O_4_, and Fe_1-x_O) become unstable with respect to structural transformations in approximately similar pressure ranges that might be related to similar shortening of Fe-O bond lengths in their structures. Hence, the resultant high-pressure polymorph of a compressed and heated iron oxide could depend on its stoichiometry, thereby suggesting chemical tuning as a route to new structural phases. In general, one could expect that a minor tuning in stoichiometry could lead either to structures with vacancies (ordered or disordered) or to modified, Fe_3_O_4_-like or Fe_2_O_3_-like high-pressure phases in new oxides. Likewise, significant shifts from known stoichiometry could potentially lead to hitherto unknown structures. For instance, the newly-discovered orthorhombic *Cmcm* polymorphs of Fe_4_O_5_^13^ and Fe_5_O_6_^14^ crystalize in structures that are linked to the high-pressure orthorhombic polymorph of Fe_3_O_4_^34^,^35^ (Fig. 4). By analogy with the existing family of calcium ferrites, 
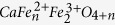
36, it was proposed that iron oxides with this *Cmcm* structure could also form such a family as 
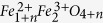
37, which includes Fe_3_O_4_, Fe_4_O_5_^13^ and Fe_5_O_6_^14^. However, the present discovery of Fe_7_O_9_ that does not belong to this family on the one hand, but having a certain structural similarity with the above oxides on the other hand (Fig. 4), suggests that the family of iron oxides that are structurally linked to the high-pressure polymorph of Fe_3_O_4_ may be more broad, e.g., like 
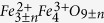
, thereby suggesting a potentially greater diversity than in the calcium ferrite oxides. For instance, Fe_9_O_11_ (n = 2) or Fe_11_O_13_ (n = 3) might be hypothetically stable under certain HP-HT conditions.

At the moment the stability field of this new Fe_7_O_9_ polymorph is not well defined, although it appears to lie at pressures higher than those of Fe_4_O_5_^13^ and Fe_5_O_6_^14^. The chemical compositions and the structural phases of iron-magnesium oxides in the Earth’s mantle remain a disputed issue that requires *in situ* investigations at HP-HT conditions under different oxygen fugacities. In this regard, the unexpected discovery of a Fe_7_O_9_ polymorph provides new insight into possible compositions of mantle phases, and provides a new type compound that may play a key role in determining physical and chemical properties.

The new oxide, Fe_7_O_9_, is a compound with a ratio of Fe^3+^/Fe^2+^ intermediate between those of Fe_3_O_4_ and Fe_4_O_5_ (Fig. 4). Both Fe_3_O_4_ and Fe_4_O_5_ are model systems for investigations of Fe^2+^–Fe^3+^ interactions in solids, demonstrating enigmatic low-temperature phase transitions of ‘metal-insulator’-type that lead to the formation of exotic ‘trimeron quasiparticles’ in Fe_3_O_4_^21^ or to even more intricate ordering patterns in Fe_4_O_5_^29^. We note that the low-temperature Verwey transition in Fe_3_O_4_ has had a strong impact on solid state physics and chemistry for decades. Thus, Fe_7_O_9_ presents an exciting compound that promises important implications for geosciences, solid state physics and chemistry with potential for industrial applications.

The recently discovered iron oxides may play important roles in the cycling of volatiles in the Earth’s deep interior^16^,^17^,^18^,^19^. For instance, oxygen could be released in the deeper part of the lower mantle via decomposition reactions of Fe_2_O_3_ and Fe_3_O_4_ into Fe_5_O_7_ and Fe_25_O_32_ above 60 GPa^17^. Further, it was recently reported that FeO_2_ could be formed in the lower mantle as a product of FeOOH goethite decomposition^18^. Moreover, this reaction could supply hydrogen to the surrounding mantle^18^. Physical and chemical properties of the newly-discovered iron oxides can therefore provide novel insights into the chemical evolution of the Earth’s interior.

## Additional Information

**How to cite this article**: Sinmyo, R. *et al*. Discovery of Fe_7_O_9_: a new iron oxide with a complex monoclinic structure. *Sci. Rep.* **6**, 32852; doi: 10.1038/srep32852 (2016).

## Supplementary Material

Supplementary Information
