# Author Correction: Discovery of Fe_7_O_9_: a new iron oxide with a complex monoclinic structure

**DOI:** 10.1038/s41598-020-62903-1

**Published:** 2020-04-15

**Authors:** Ryosuke Sinmyo, Elena Bykova, Sergey v. Ovsyannikov, Catherine McCammon, Ilya Kupenko, Leyla Ismailova, Leonid Dubrovinsky

**Affiliations:** 1 Bayerisches Geoinstitut, Universität Bayreuth, D-95440 Bayreuth, Germany; 2 Laboratory of Crystallography, Universität Bayreuth, D-95447 Bayreuth, Germany; 3 European Synchrotron Radiation Facility, BP220, F-38043 Grenoble, France

Correction to: *Scientific Reports* 10.1038/srep32852, published online 08 September 2016

Table S3 is missing from the supplementary material accompanying this Article and is provided below.

## Supplementary information

Table S3.

