# Supplementary material for: Discovery of Fe7O9: a new iron oxide with a complex monoclinic structure
Source: Sci Rep. 2016 Sep 8;6:32852. doi: 10.1038/srep32852 (PMC5015080; doi:10.1038/srep32852)
Supplement: Supplementary Information [file srep32852-s1.pdf]

Supplementary information for

Discovery of Fe_7O_9: a new iron oxide with a complex monoclinic structure

Ryosuke Sinmyo[1,*], Elena Bykova[1], Sergey V. Ovsyannikov[1], Catherine McCammon[1], Ilya Kupenko[2], Leyla Ismailova[1], and Leonid Dubrovinsky[1]

[1]*Bayerisches Geoinstitut, Universität Bayreuth, D-95440 Bayreuth, Germany*

[2]*Europian Synchrotron Radiation Facility, BP220, F-38043 Grenoble, France*

* Corresponding author (E-mail: ryosuke.sinmyo@elsi.jp). *Now at Earth-Life Science Institute, Tokyo Institute of Technology, 152-8550 Tokyo, Japan*

METHODS

Preparation of samples. For synthesis of Fe_7O_9, and Mg-doped Fe_7O_9 we used three different starting mixtures including: (*i*) Fe metal + Fe_3O_4 magnetite, (*ii*) Fe metal + Fe_2O_3 (95% enriched in ^{57}Fe) hematite, and (*iii*) MgO + Fe_2O_3 (95% enriched in ^{57}Fe) hematite with a bulk composition of $MgFe^{3+}{}_2O_4$. All the mixtures were well ground and dried before the syntheses. We used a Kawai-type multi-anvil press to generate high pressures (*38*). We found the optimal conditions for synthesis of these Fe_7O_9 polymorphs to be as follows: 24-26 GPa and 1873-1973 K. In these syntheses the starting material was compacted in an Au capsule that was inserted inside a double cylinder, where the internal part was a MgO insulating layer, and the external part was a $LaCrO_3$ heater. These parts were then assembled with a W3%Re-W25%Re thermocouple inside an octahedron-shaped pressure medium. All procedures are similar to those reported earlier (*38*).

Chemical and structural characterization of samples. Both $(Mg.Fe)_7O_9$ and Fe_7O_9 samples were removed from the capsules and characterized using chemical and structural methods. The chemical analysis was performed by means of electron microprobe analysis using a JEOL JXA-8200 under operating conditions of 15 kV and 15 nA. The Mg/Fe ratio was determined for $(MgFe)_7O_9$ by electron microprobe analysis, while the Fe_7O_9 sample was not analyzed by microprobe. Oxygen was not quantified during the analysis of $(MgFe)_7O_9$. Electron microprobe data showed the Mg/Fe homogeneity of the samples, and, in particular, permitted the amount of incorporated Mg at the cation sites in $(Mg,Fe)_7O_9$ to be constrained as 15.1±0.3 at.%. Hence, Mg-doped samples had a composition of $Mg_{1.06}Fe^{2+}{}_{1.94}Fe^{3+}{}_{4.00}O_9$. We observed (Mg,Fe)O ferropericlase co-existing with the $(Mg,Fe)_7O_9$ phase by scanning electron microscope analysis. Single crystal X-ray diffraction studies were carried out on a 3-circle Bruker diffractometer equipped with a SMART APEX CCD detector and a high-brilliance Rigaku rotating anode (Rotor Flex FR-D, Mo-Kα radiation) with Osmic focusing X-ray optics. Extraction of the reflection intensities was performed with CrysAlisPro software (Agilent Technologies UK Ltd.). The crystal structure was solved by a direct method and refined by a full matrix least-squares method in an anisotropic approximation for metal atoms using SHELXS and SHELXL software (*39*), respectively (Table S1). The crystal structure was visualized using the VESTA 3 software package (*40*). The X-ray crystallographic coordinates for structures of Fe_7O_9 and $(Mg,Fe^{2+})_3Fe^{3+}{}_4O_9$ have been deposited at the Inorganic Crystal Structure Database (ICSD) under deposition number CSD 430601 and 431513, respectively. These data can be obtained free of charge from FIZ Karlsruhe, 76344 Eggenstein-Leopoldshafen, Germany (fax: (+49)7247-808-666; e-mail: crysdata@fiz-karlsruhe.de) through the hyperlink "https://www.fiz-karlsruhe.de/en/leistungen/kristallographie/kristallstrukturdepot/order-form-request-for-deposited-data.html".

Mössbauer spectroscopy. The electronic states of iron in Fe_7O_9 and $(Mg,Fe)_7O_9$ samples were investigated by means of Mössbauer spectroscopy using a synchrotron Mössbauer source (SMS). For these studies we used the same single crystals of Fe_7O_9 and $(Mg,Fe)_7O_9$ that were studied using X-ray diffraction. The SMS spectra were collected on the Nuclear Resonance ID18 Beamline of the European Synchrotron Radiation Facility,

ESRF (Grenoble, France) (*41*) using the (111) Bragg reflection of a $^{57}FeBO_3$ single crystal mounted on a Wissel velocity transducer driven with a sinusoidal wave form (*42*). The synchrotron X-ray beam was focused to 20 μm vertical and 10 μm horizontal dimensions using Kirkpatrick-Baez mirrors. The linewidth of the SMS and absolute position of the centre shift (CS) were controlled before and after each measurement using a $K_2Mg^{57}Fe(CN)_6$ reference single line absorber. The velocity scale was calibrated using a 25 μm thick natural α-Fe foil. The typical acquisition time for each spectrum was about 5.5 hours. All spectra were fitted using a full transmission integral with a normalized Lorentzian-squared source lineshape using the MossA software package (*43*).

References

[38] H. Keppler, D. J. Frost, in *EMU Notes in Mineralogy,* R. Miletich Ed. (Eotvos University Press, Budapest, 2005), p. 1-30.

[39] G. M. Sheldrick, A short history of SHELX. *Acta Crystallogr. Sect. A* **64**, 112-122 (2008).

[40] K. Momma, F. Izumi, VESTA 3 for three-dimensional visualization of crystal, volumetric and morphology data. *J. Appl. Crystallogr.* **44**, 1272-1276 (2011).

[41] R. Rüffer, A. I. Chumakov, Nuclear resonance beamline at ESRF. *Hyperfine Interact.* **97/98**, 589-604 (1996).

[42] V. Potapkin, et al., Effect of iron oxidation state on the electrical conductivity of the Earth’s lower mantle. *Nat. Commun.* **4**, 1427 DOI: 10.1038/ncomms2436 (2012).

[43] C. Prescher, C. McCammon, L. Dubrovinsky, MossA: a program for analyzing energy-domain Mossbauer spectra from conventional and synchrotron sources. *J. Appl. Crystallogr.* **45**, 329-331 (2012).

Supplementary Figure 1

**$(Mg,Fe^{2+})_3Fe^{3+}{}_4O_9$**	**Fe_7O_9**
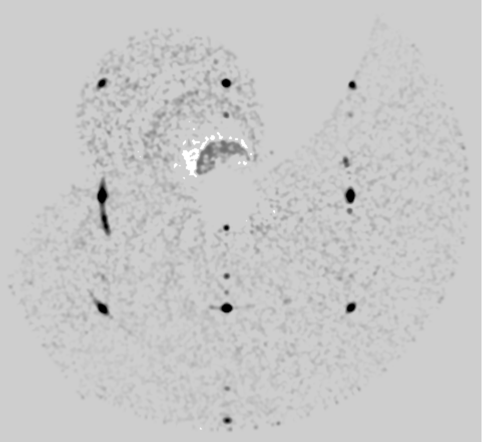	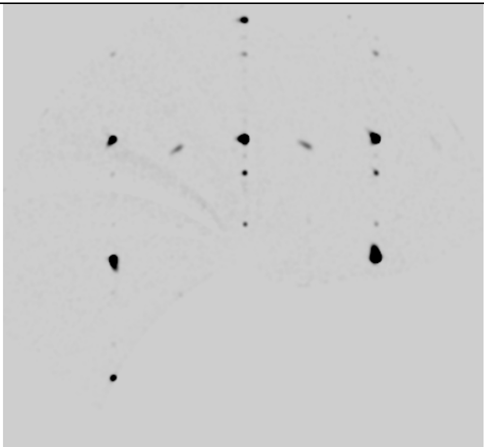

Projections of the X-ray diffraction data in reciprocal space for 0*kl* plane. The diffraction images of Fe_7O_9 (left) and $(Mg,Fe^{2+})_3Fe^{3+}{}_4O_9$ (right). We observed signs of diffuse scattering mainly located around reflections in *hkl* (h = 0,1,2…) planes. This suggests defects in the structures which in turn would influence the shape of anisotropic parameters. In $(Mg,Fe^{2+})_3Fe^{3+}{}_4O_9$ the effect was stronger than in Fe_7O_9.

TABLE S1. Unit cell parameters and refinement parameters.

	Fe_7O_9	$(Mg,Fe)_3Fe_4O_9$
a (Å)	9.696(2)	9.6901(12)
b (Å)	2.8947(6)	2.8943(5)
c (Å)	11.428(3)	11.4397(15)
ß (°)	101.69(2)	102.045(14)
Reflections collected	631	985
Density (g/cm^3)	5.656	5.308
R_{int}	0.0288	0.0169
Independent reflections (all)	631	438
Independent reflections (I ≥2σ(I))	624	371
R_1 (all data)	0.0345	0.0508
R_1 (I ≥2σ(I))	0.0339	0.0427
wR_2 (all data)	0.0907	0.1229
wR_2 (I ≥2σ(I))	0.0898	0.1178
Goodness-of-fit on F^2	1.187	1.194
Mg (cation per nine O)	0	1.06
Fe (cation per nine O)	7	5.94

TABLE S2. Refined fractional occupancies, refined atomic coordinates and equivalent isotopic temperature factor.

	x	y	z	U_{eq}	X_{Mg}	X_{Fe}
Fe_7O_9						
Fe1	0.5000	0.0000	0.5000	0.0080(4)	0	1
Fe2	0.27286(11)	0.0000	0.63323(9)	0.0079(3)	0	1
Fe3	0.35412(10)	0.0000	0.93362(9)	0.0073(3)	0	1
Fe4	0.06979(10)	0.0000	0.78819(11)	0.0104(3)	0	1
O1	0.4131(5)	0.0000	0.7794(5)	0.0088(10)		
O2	0.0000	0.0000	0.0000	0.0083(15)		
O3	0.1300(6)	0.0000	0.4503(5)	0.0081(10)		
O4	0.3663(5)	0.0000	0.3401(5)	0.0075(11)		
O5	0.2986(5)	0.0000	0.1005(5)	0.0074(10)		
$(Mg,Fe)_3Fe_4O_9$						
Fe1	0.5000	0.0000	0.5000	0.0079(7)	0.259(12)	0.741(12)
Fe2	0.27221(12)	0.0000	0.63205(10)	0.0093(5)	0.103(8)	0.897(8)
Fe3	0.35310(12)	0.0000	0.93359(11)	0.0088(5)	0.072(9)	0.928(9)
Fe4	0.06782(13)	0.0000	0.78759(14)	0.0137(5)	0.226(8)	0.774(8)
O1	0.4108(5)	0.0000	0.7793(5)	0.0098(12)		
O2	0.0000	0.0000	0.0000	0.0147(19)		
O3	0.1287(5)	0.0000	0.4501(5)	0.0097(12)		
O4	0.3654(6)	0.0000	0.3398(5)	0.0098(12)		
O5	0.2990(5)	0.0000	0.1007(5)	0.0099(13)		
