# Supplementary material for: Author Correction: Discovery of Fe7O9: a new iron oxide with a complex monoclinic structure
Source: Sci Rep. 2020 Apr 15;10:6622. doi: 10.1038/s41598-020-62903-1 (PMC7160142; doi:10.1038/s41598-020-62903-1)
Supplement: Supplementary file 1 — Table S3. [file 41598_2020_62903_MOESM1_ESM.pdf]

TABLE S3. Hyperfine parameters obtained from Mössbauer spectroscopy.

	Fe_7O_9	$(Mg,Fe)_3Fe_4O_9$
1st component		
Center shift (mm/s)	0.559(3)	0.47(2)
Quadrupole splitting (mm/s)	0.337(4)	0.40(4)
FWHM	0.259(7)	0.70(6)
Abundance (%)	73.6(7)	78(2)
2nd component		
Center shift (mm/s)	0.954(3)	0.97(1)
Quadrupole splitting (mm/s)	2.739(6)	2.84(3)
FWHM	0.174(10)	0.31(4)
Abundance (%)	26.4(7)	22(2)
